# Implementation of the Gamut of Physiotherapy Maneuvers in Restoration and Normalization of Functional Potencies in a Patient With a Hemorrhagic Stroke: A Case Report

**DOI:** 10.7759/cureus.33035

**Published:** 2022-12-28

**Authors:** Nikita Bhusari, Vikrant G Salphale, Nishigandha P Deodhe

**Affiliations:** 1 Department of Neuro Physiotherapy, Ravi Nair Physiotherapy College, Datta Meghe Institute of Medical Sciences, Wardha, IND

**Keywords:** strength, motor, recovery, brunstorm, hemorrhage, roods, decompressive craniotomy, tone, intracranial bleed, stroke

## Abstract

A stroke is a medical emergency characterized by a sudden onset of focal neurological deficits due to an interruption in the blood flow to the brain tissues, with signs and symptoms persisting for more than 24 hours. Motor, sensory, recognition, language, and perceptual deficiencies are typical signs of the disease, depending on the areas affected, the size of the injury, and the origin of the injury. Patients who have had a stroke frequently have problems like weakness, stiffness, and altered movement patterns in addition to poor balance and mobility issues. Numerous physiotherapeutic strategies concentrate on helping stroke victims recover quickly. Stroke-related mortality rates have decreased over the past few decades due to advancements in stroke therapy and rehabilitation. One approach that can be primarily used to normalization of tone is facilitation by Rood’s technique. The present case report is of a 45-year-old female with a history of hypertension presented with complaints of weakness on the right side of the body. The patient had right hemiplegia with more involvement of the right upper extremity. The patient underwent a decompressive craniotomy. On investigation, the magnetic resonance imaging (MRI) report revealed an area of blood density attenuation with multiple air foci in the left gangliocapsular region. Treatment was started after the patient was operated on. An approach-oriented rehabilitation program was planned for the patient. Physiotherapy maneuvers such as the proprioceptive neuromuscular facilitation (PNF) approach and Rood’s approach were used to restore and normalize functional potencies and recover the patient’s condition. Oral facial facilitation was also used for swallowing frequency control, sensory awareness, and motor control. Posttreatment changes such as changes in muscle tone, strength, and mobility, which are essential for patients with the activity of daily living (ADLs), were observed. Outcome measures used in this patient are the Functional Independence of Measures (FIM) scale, Brunnstrom grading, voluntary control grading, and the National Institute of Health Stroke Scale (NIHSS).

## Introduction

Intracranial hemorrhage (ICH) is the term used to describe any bleeding that takes place inside the intracranial vault, which comprises the brain parenchyma and surrounding meningeal spaces. Epidural hemorrhage, subdural hemorrhage, subarachnoid hemorrhage, and intraparenchymal hemorrhage are the four main kinds of intracranial bleeding. It can be difficult to differentiate between ICH and an ischemic stroke in acute presentation [1]. The symptoms of an ICH rather than an ischemic stroke include coma, headache, vomiting, seizures, neck stiffness, and high diastolic blood pressure (BP), but only neuroimaging can make a definitive diagnosis [2]. Stroke patients must first improve their general physical condition and fundamental daily living skills before improving their hand movement and hand muscle strength for instrumental activities of daily living training [3]. Similar to ICH without an underlying lesion, there is typically little evidence to recommend surgically removing the hemorrhage [4].

Reflex-inhibiting movement patterns by Bobath assure that normal tone is maintained in the affected limb and the development of abnormal movement patterns is prevented [5]. She also says that for complete rehabilitation, incorporating the affected upper limb into all everyday tasks is essential. Unfortunately, like with all motor remediation theories, empirical data supporting the use of this approach is still inconclusive. When a blood artery inside the brain or between the skull and brain bursts, it causes an intracranial hematoma. The brain tissue is compressed by blood accumulation (hematoma). Brain tissue is compressed by the hematoma, a blood clump. A cerebral hematoma is potentially life-threatening and frequently requires rapid treatment, but some head injuries, such as those that merely result in momentary loss of consciousness may be minor.

CT scans are extremely useful for diagnosing hematomas. Generally, clinicians assume that progressive loss of consciousness associated with a head injury can be attributed to bleeding in the skull. In this latter group, the majority of the study on the advantages of surgery has been conducted [6]. To remove the blood, surgery is frequently but not always necessary for cerebral hematomas. In a decompressive craniectomy (DC), a section of the skull is cut away to provide space for enlarged brain tissue. Although it has been used for many years to manage patients with brain edema and/or intracranial hypertension, its current use in therapy is debatable. Patients with malignant middle cerebral artery stroke have higher survival rates after DC, but some of them still experience moderate-to-severe impairment. When treating individuals with diffuse traumatic brain damage, an early (neuroprotective) DC is not more effective than standard medical care [6]. Stroke places a tremendous burden on health resources worldwide [7]. The incidence of stroke is currently substantially greater in India than in Western industrialized nations [8]. In stroke, only a few possible symptoms include headache, nausea, convulsions, and localized or generalized neurologic problems. Deep structures are more prone to experience hypertension-related ICH, and as BP increases, the risk of ICH increases as well. Other risk factors for ICH include heredity, alcohol use, and low total serum cholesterol levels. Due to stroke, the patient's quality of life gets affected as it has a direct impact on health systems; serious disabilities and functional limitations lead to compromised quality of life [9].

## Case presentation

Patient information

The 45-year-old female patient came to the hospital with flaccidity of the right upper and lower limbs. Bed mobility was very challenging for her. There were no sensory issues or deformities. Previously, she was affected by strokes twice, for which she underwent medical and physiotherapy treatment in the early stages. She had been taking medicine for her hypertension for the past two years. Now, after two years, she was affected by the same condition and got admitted in July for further treatment. On investigation, the magnetic resonance imaging (MRI) revealed an area of blood density attenuation with multiple air foci in the left gangliocapsular region. An intracranial hemorrhage was observed on a CT scan of the brain. In this case, she underwent a decompressive craniotomy. After 36 hours of monitoring in the intensive care unit (ICU), she was shifted to the surgery ward for further management.

Clinical findings

In terms of time, place, and people, the patient was orientated. The vital signs were as follows: temperature 95.5 °F, BP 130/79 mmHg, pulse 63 beats per minute, respiration rate 20 breaths per minute, and oxygen saturation 98%. On neurological assessment, the Glasgow Coma Scale (GCS) scored 7 as severe. A score of 30 was also determined as severe by the NIHSS. According to the modified Rankin scale (mRS), the patient scored 5. The affected right upper limb and right lower limb were supported with a pillow. Ryle’s tube was attached for feeding. The patient was on intermittent catheterization as the bowel and bladder functions were hampered. In Table 1, the results of the deep tendon reflexes are presented.

**Table 1:** Grading of deep tendon reflexes.

Deep tendon reflex	Grading			
	Day 1	Day 7	Day 14	Day 21
Jaw jerk	2+	2+	3+	2+
Biceps jerk	2+	2+	3+	2+
Supinator jerk	2+	2+	3+	2+
Triceps jerk	2+	3+	2+	2+
Knee jerk	1+	1+	2+	2+
Ankle jerk	0	1+	1+	2+
Plantar response	Mute	Mute	Extensor	Extensor

On day 7, we noticed a change in the tone of the patient's right upper and lower limbs, so we evaluated them using the Modified Ashworth Scale (MAS). Table 2 presents the findings.

**Table 2:** Tone assessment.

Tone	Left	Right
Upper limb	Normal	Flaccid (Grade 0)
Lower limb	Normal	Hypotonia (Grade 2)

Diagnostic methods

**Figure 1:**
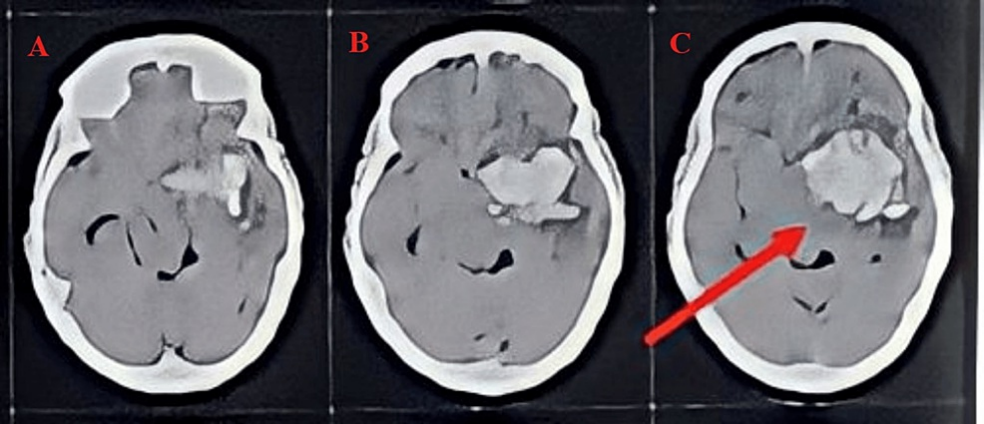
Computed tomography scan showing intracranial bleed. (A) Bony defects on the right frontal, parietal, and temporal regions. (B) Hemorrhage in the gangliocapsular region. (C) Midline shift of 9 mm.

The patient underwent an investigation of a CT scan (Figure 1), which revealed an area of blood density attenuation (+60 to +70 HU), with multiple air foci in the left gangliocapsular region resolving hemorrhage with mass effect in the form of effacement of adjacent solo gyral spaces. Corpus callosum lipoma was noted. A midline shaft of 9 mm was noted toward the left. Bony defects in the right frontal, parietal, temporal, and occipital bones on the right with overlying soft tissue swelling and sutures were seen.

Diagnostic assessment

Intervention

**Figure 2:**
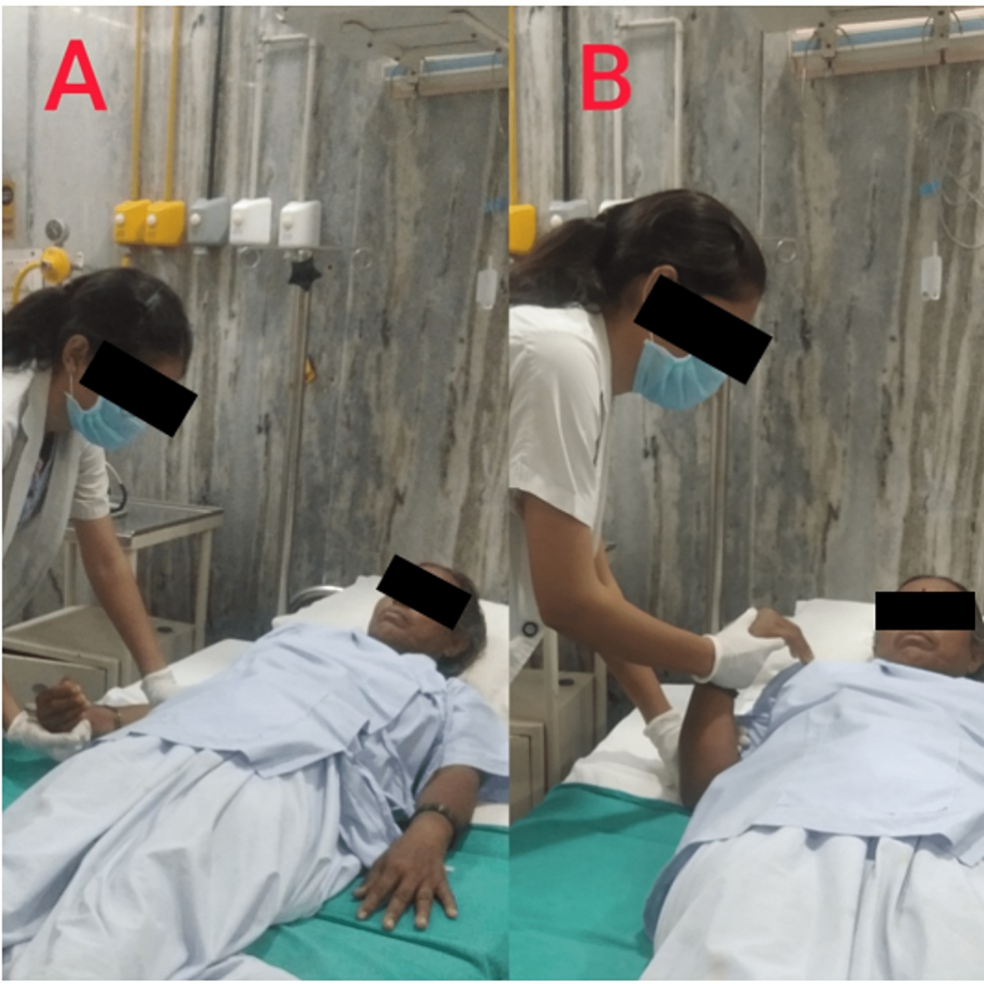
Passive range of motion of the right upper limb. (A) Extended elbow. (B) Flexed elbow.

Phase I (day 1 to four weeks): Roods method - facilitating treatments used for treating flaccidity in the right upper limb include quick brushing, quick icing of the muscular bellies, tapping, and performing severe joint compression. Spasticity was treated with upper limb inhibitory techniques such as slow stroking and rolling, prolonged stretching, pressure on the muscle insertion, light joint compression, and unresisted contractions. It was advised to practice breathing exercises throughout the day as often as possible. These include glossopharyngeal breathing and pursed-lip breathing. On the patient, passive movement of the right upper limb was performed, as shown in Figure 2. The patient was instructed in bed mobility exercises for supine to side lying, such as logrolling and segmental rolling with additional support. Oral facial facilitation helped with swallowing frequency control, sensory awareness, and motor control. The tone and swallow reflex were improved by applying ice to the platysma and face muscles for 5-20 minutes. Tone improvement was seen with vibration; oral awareness was improved using manipulation techniques such as tapping, stroking, patting, and delivering intense pressure directly to muscles with the fingertips. Oral motor sensory exercises were done to enhance chewing and slurring abilities, such as lip closure, tongue movement, and deglutition. Practicing good skin care is very essential, such as giving patients a sponge bath twice daily and then cleaning their skin afterward and applying a moisturizer or lotion with humectants as the main ingredient to prevent skin dryness.

**Figure 3:**
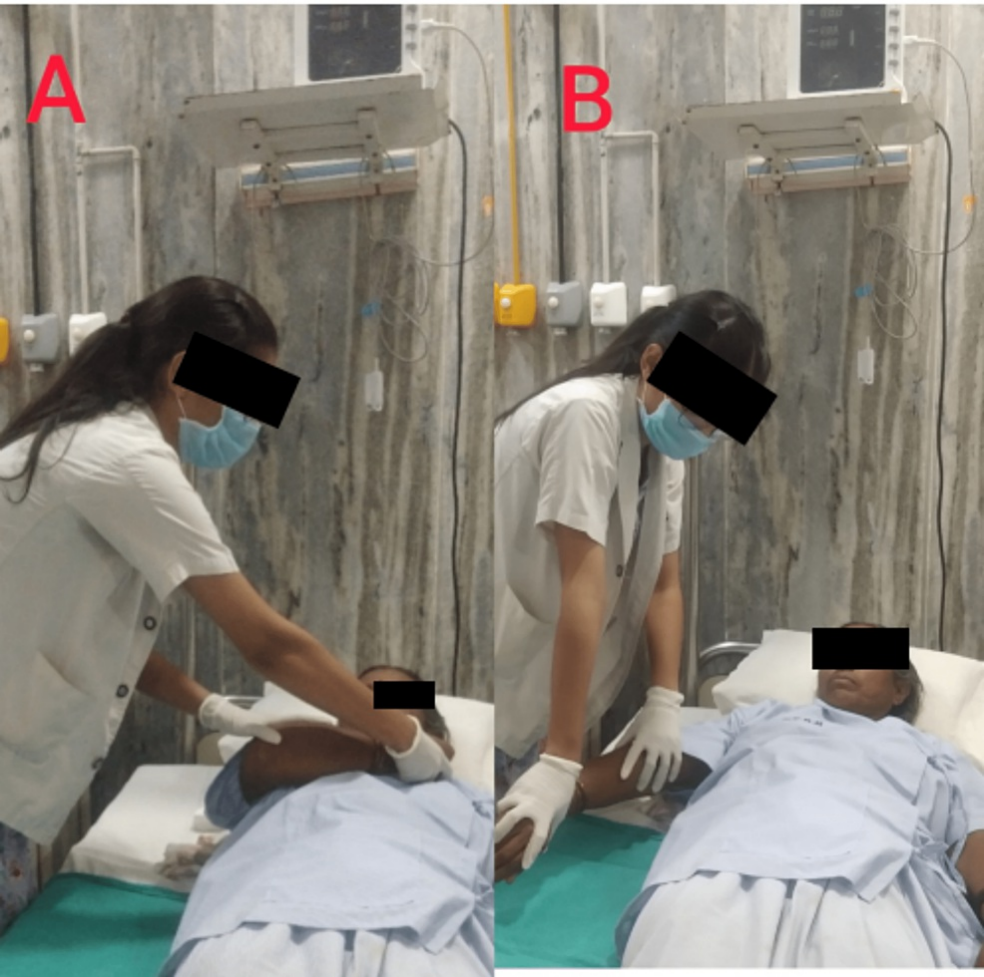
Shoulder proprioceptive neuromuscular facilitation pattern D1. (A) The starting position is shoulder flexion, adduction, external rotation, forearm supination, wrist flexion, and finger flexion. (B) The ending position is shoulder extension, abduction, external rotation, forearm pronation, and wrist and finger extension.

Phase II (four to eight weeks): Roods Method - fast brushing, quick icing of the muscular bellies, tapping, and severe joint compression are facilitative treatments for relieving flaccidity in the lower limbs. Spasticity was treated with upper limb inhibitory techniques such as soft stroking and rolling, prolonged stretching, pressure on the muscle insertion, mild joint compression, and unresisted contractions. The proprioceptive neuromuscular facilitation (PNF) technique was used to improve the physical functioning of the patient (Figure 3). It was practiced as often as the patient can during the day in between each round of breathing exercises. Inhaling via the mouth and exhaling through a pursed lip are two of these breathing exercises. The patient was shown how to move around in bed segmentally to transition from supine to side lying. Oral face facilitation was performed to aid in frequency control of swallowing, sensory awareness, and motor control; 15-25 minutes of platysma muscle freezing to enhance tone. Vibration can enhance tone, and manipulation techniques like tapping, stroking, patting, and applying force directly to muscles with the fingertips can improve oral awareness. Oral motor sensory drills were done to improve deglutition, tongue movement, and lip closure when chewing and slurring. A moisturizer or lotion with humectants is used to prevent skin dryness.

Outcome measures

Table 3 lists the various scales that were used to assess the patient's posttreatment changes.

**Table 3:** Findings of outcome measures.

Serial no.	Scales	Pretreatment	Posttreatment
1	Brunnstrom stage of recovery	Upper limb stage 1, lower limb stage 1	Upper limb stage 5, lower limb stage 5
2	National Institute of Health Stroke Scale	30/42	12/42
3	Voluntary Control Grading	Grade 0	Grade 2
4	Functional Independence Measure	70/126	95/126
5	Modified Rankin Scale (mRS)	5/6	1/6

## Discussion

The recovery process following a stroke varies from individual to individual. It is difficult to estimate how many abilities an individual will be able to regain and when they will be able to do so. Stroke survivors frequently experience lifelong issues as a result of brain damage. Before regaining their former freedom, some people require extensive rehabilitation, while many others never fully recover and require continual help. A successful rehabilitation outcome seems to be closely related to high patient satisfaction, motivation, and involvement. Rehabilitation plays a major role in recovery in stroke patients [10]. Rehabilitation aims to enable patients to live independently and achieve functional recovery through activities of daily living; specific rehabilitation objectives may differ for different people. The majority of research has discovered that regular motor practice and real-world motor training are helpful for stroke patients' motor recovery [11]. Enhancing postural control, motor function, and the patient's self-perception and comprehension of the risk of falls are the primary goals of stroke recovery, which improve the patient's capacity to operate safely in a dynamic environment [12]. For the patient to achieve safety, independence, and effective movement, rehabilitation is necessary. Successful therapeutic strategies reduce problems and enhance patient outcomes [13]. The strengths and needs of each client can be quickly identified, and effective strategies and education for clients and their families can be put into place when the client's mobility is assessed early on.

Proprioceptive facilitation techniques for the upper limb that involve performing joint approximation 10 times with a 30-second hold during therapy have proven effective [14]. In a hemiplegic arm, recovery typically occurs in a proximal to distal gradient, with finger movements returning last or not at all and shoulder or elbow movements returning earliest [15]. Several changes were observed following treatment, including changes in muscle tone, strength, and mobility, which are crucial for patients with ADLs. To improve the static and dynamic balance after a stroke, several protocols are focused on gait and balance abnormalities, which are a substantial handicap and the leading cause of falls with sometimes severe consequences. Hemiplegic gait is a combination of abnormalities and compensatory movements caused by poststroke residual functions. To improve gait after a stroke, balancing, neurophysiological, and motor learning therapies are all used.

## Conclusions

The prognosis of intracranial bleeding is good, and the change in the patient's quality of life is enhancing. Physiotherapy treatment that is individualized to the patient’s requirements and objectives and focuses on managing symptoms, enhancing function, and involvement helps people to live their lives with quality. The physiotherapeutic interventions used in this case were very effective, and the recovery rate was quite good, which improved the patient's quality of life and strength. 
